# Coronavirus spike protein-specific antibodies indicate frequent infections and reinfections in infancy and among BNT162b2-vaccinated healthcare workers

**DOI:** 10.1038/s41598-023-35471-3

**Published:** 2023-05-24

**Authors:** Pekka Kolehmainen, Moona Huttunen, Alina Iakubovskaia, Sari Maljanen, Sisko Tauriainen, Emrah Yatkin, Arja Pasternack, Rauno Naves, Laura Toivonen, Paula A. Tähtinen, Lauri Ivaska, Johanna Lempainen, Ville Peltola, Matti Waris, Laura Kakkola, Olli Ritvos, Ilkka Julkunen

**Affiliations:** 1 Institute of Biomedicine, University of Turku, Turku, Finland; 2 Department of Physiology, Biomedicum, University of Helsinki, Helsinki, Finland; 3 Central Animal Laboratory, University of Turku, Turku, Finland; 4 Department of Paediatrics and Adolescent Medicine, Turku University Hospital and University of Turku, Turku, Finland; 5 InFLAMES Research Flagship Center, University of Turku, Turku, Finland; 6 Clinical Microbiology, Turku University Hospital, Turku, Finland

**Keywords:** SARS-CoV-2, RNA vaccines, Antibodies

## Abstract

The prevalence of seasonal human coronavirus (HCoV) infections in early childhood and adults has not been well analyzed in longitudinal serological studies. Here we analyzed the changes in HCoV (229E, HKU1, NL63, OC43, MERS, and SARS-CoV-2) spike-specific antibody levels in follow-up serum specimens of 140 children at the age of 1, 2, and 3 years, and of 113 healthcare workers vaccinated for Covid-19 with BNT162b2-vaccine. IgG antibody levels against six recombinant HCoV spike subunit 1 (S1) proteins were measured by enzyme immunoassay. We show that by the age of three years the cumulative seropositivity for seasonal HCoVs increased to 38–81% depending on virus type. BNT162b2 vaccinations increased anti-SARS-CoV-2 S1 antibodies, but no increase in seasonal coronavirus antibodies associated with vaccinations. In healthcare workers (HCWs), during a 1-year follow-up, diagnostic antibody rises were seen in 5, 4 and 14% of the cases against 229E, NL63 and OC43 viruses, respectively, correlating well with the circulating HCoVs. In 6% of the HCWs, a diagnostic antibody rise was seen against S1 of HKU1, however, these rises coincided with anti-OC43 S1 antibody rises. Rabbit and guinea pig immune sera against HCoV S1 proteins indicated immunological cross-reactivity within alpha-CoV (229E and NL63) and beta-CoV (HKU1 and OC43) genera.

## Introduction

Of the seven human coronaviruses (HCoVs) identified to date, SARS-CoV-2 and the four seasonal coronaviruses, 229E, HKU1, NL63, and OC43, are endemic worldwide causing variable morbidity in different populations. Middle East Respiratory syndrome virus (MERS) is endemic in limited geographic areas such as the Arabic peninsula and SARS after causing a relatively worrisome epidemic in many countries has been extinct since 2003. Seasonal coronaviruses have been estimated to cause 15–30% of the upper respiratory tract infections^1^, with mild symptoms in the majority of cases, although severe pediatric respiratory infections can occur^2–4^.

Maternal antibodies may provide immune protection against viral infections during the first six months of life^5,6^. After the disappearance of maternal antibodies, at the time of increasing human contacts e.g. in the daycare environment, the infants are more susceptible to respiratory virus infections such as those caused by seasonal HCoVs. The prevalence of HCoV infections in early childhood has not been very well characterized. Later in life, during adolescence and adulthood, most individuals are likely (re)exposed to seasonal HCoVs, which leads to most adults having persistent antibody levels against the different HCoVs^7,8^. SARS-CoV-2 infections induce the production of antibodies especially against SARS-CoV-2 spike protein (S), nucleoprotein (N), or both^9,10^. The majority of serological studies on seasonal HCoVs have focused on the presence of anti-N antibodies^8,11–14^. However, a more comprehensive and specific picture on the rate of HCoV infections and reinfections is obtained by analyzing the presence of anti-S antibodies. Cross-protection of pre-existing anti-seasonal HCoV antibodies against SARS-CoV-2 has been under an intensive discussion^15–17^. Also, the potential role of COVID-19 vaccination in the protection against seasonal HCoV infections has remained uncharacterized.

Here we describe the seropositivity rates and IgG antibody levels against HCoV spike subunit 1 (S1) proteins in sequentially collected serum samples of Finnish children, and in BNT162b2-vaccinated healthcare workers (HCWs) coupled with data on RT-PCR-confirmed circulation of HCoVs in the community. Our data demonstrate increasing seropositivity for HCoV S1-binding IgG antibodies in early childhood, and a good correlation of these antibodies with the corresponding nucleoprotein (N) binding IgG antibodies. Changes in HCoV antibody levels in the follow-up of HCWs match well with the data on circulating HCoVs. BNT162b2-vaccination has no effect on anti-S1 antibody levels against seasonal HCoVs or MERS.

## Results

Based on the overall lower amino acid sequence identity (Supplementary Table 1) and on our previous work with SARS-CoV-2^10^, spike protein subunit 1 (S1) was selected as the antigen to set up an enzyme immunoassay (EIA) for the detection of HCoV S binding IgG antibodies. S1 subunits were produced as mFc-fusion (S1-mFc) proteins in HEK-293F cells, purified (Supplementary Fig. 1) and used in EIA.

### Seropositivity and HCoV spike-specific IgG antibody levels in children of 1 to 3 years of age

**Figure 1:**
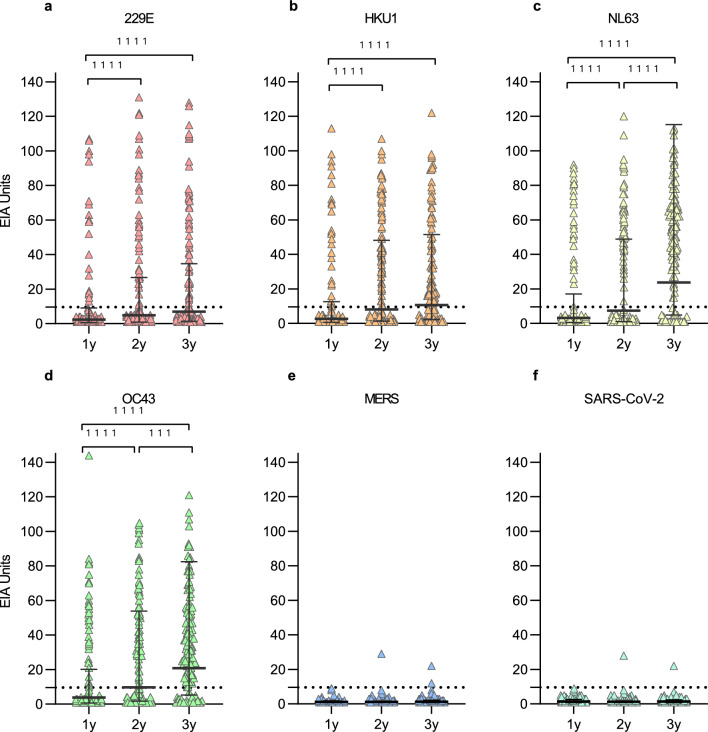
HCoV S1-specific IgG antibody responses in children at 1, 2 and 3 years of age. HCoV S1-specific IgG antibody levels were measured with EIA from sera collected from 140 children at 1, 2, and 3 years of age, during the years 2009 to 2013. Geometric means and geometric standard deviations of antibody levels are shown. Differences between the groups were analyzed using Wilcoxon matched pairs test. Two-tailed P-values < 0.05 were considered statistically significant. P-values are < 0.0001 (1111) and 0.0004 (111). Dashed line indicates the cutoff value for seropositivity.

Serum specimens collected in 2009–2013 from 140 healthy children (74 male and 66 female, Table 1) at 1, 2, and 3 years of age were analyzed by EIA for antibodies against S1 proteins of four seasonal HCoVs (229E, HKU1, NL63, OC43), MERS and SARS-CoV-2. The absorbance values were converted to EIA units enabling reliable identification of seropositive samples and to compare antibody levels against different coronavirus species (Fig. 1). An increase in the geometric mean antibody levels between 1- and 2-year samples was significant for all four seasonal HCoVs (p < 0.0001) while the change between 2 and 3 years was significant only for NL63 and OC43 (p < 0.0001 and p = 0.0004, respectively). One participant had low levels of MERS and SARS-CoV-2 S1-binding IgG antibodies at 2 and 3 years of age.

**Table 1:** Characteristics of the study cohorts and serum sampling intervals.

	Children	COVID-19 vaccinated HCWs
N	140	113
Female (%)	66 (47%)	104 (92%)
Male (%)	74 (53%)	9 (8%)

	Months	Years
Age 1st sampling (mean and range)	13.7 (11.6–16.6)	43 (25–65)
Age 2nd sampling (mean and range)	25.3 (23.8–27.4)	
Age 3rd sampling (mean and range)	37.5 (36.1–39.6)	

Sampling interval	Months	Months
Time between 1st and 2nd sample	11.6 (7.8–14.7)	2.3 (1.3–6.2)
Time between 2nd and 3rd sample	12.1 (10.3–14.6)	8.5 (7.5–10.5)

The rate of antibody positive children increased by age for the four seasonal HCoVs and the antibody levels remained elevated in most of the seropositive children (Table 2). In all age groups the seropositivity was the highest for OC43 with a seropositivity of 31% in 1-year-olds and 81% in 3-year-olds. At 3 years of age the seropositivity rate for 229E (37%) was lower than for the other seasonal HCoVs (59% for HKU1, 76% for NL63, and 81% for OC43). It is noteworthy that the cumulative seropositivity for HKU1 declined between 2 and 3 years of age, while in 229E, NL63 and OC43 the annual and cumulative seropositivity rates increased almost at the same rate (Table 2). A decline in anti-HKU1 S1 antibodies was observed in previously seropositive children whose sequential samples showed a decrease in geometric mean IgG antibody levels (GMALs) from 2 to 3 years (44 to 33 EIA units). For 229E, NL63, and OC43 the mean IgG antibody levels in seropositive children remained relatively stable during the 3-year follow-up. Gender had no significant effect on the HCoV S1 antibodies in the studied children at any time point (Supplementary Fig. 2).

**Table 2:** Seroprevalence of IgG antibodies against HCoV S1 in 140 children at ages of 1, 2, and 3 years.

	Age	Seropositivity		Cumulative seropositivity		Antibody level in seropositive (EIA units)		Reinfection rate	
		n	%	n	%	GMAL ± SD	CI 95%	n	%
229E	1y	20	14	20	14	43 ± 2.1	30–61	–	–
	2y	44	31	45	32	46 ± 2.0	37–57	5	25
	3y	52	37	53	38	45 ± 2.0	37–55	11	24
HKU1	1y	29	21	29	21	41 ± 2.1	31–55	–	–
	2y	69	49	73	52	44 ± 1.9	37–51	4	14
	3y	83	59	93	66	33 ± 2.0	29–39	6	8
NL63	1y	34	24	34	24	54 ± 1.6	45–64	–	–
	2y	63	45	64	46	55 ± 1.5	49–61	7	21
	3y	107	76	108	77	54 ± 1.5	50–59	11	17
OC43	1y	43	31	43	31	39 ± 1.8	32–47	–	–
	2y	76	54	79	56	42 ± 1.8	37–48	14	33
	3y	114	81	114	81	38 ± 1.8	34–42	15	19
Any HCoV	1y	88	63	88	63	44 ± 1.9	39–49	–	–
	2y	119	85	120	86	46 ± 1.8	43–49	27	31
	3y	137	98	137	98	42 ± 1.9	39–45	40	34

The table presents the seroprevalence and cumulative seroprevalence in numbers (n) and percentages (%) of positive individuals in each age group. The geometric mean titers (GMT) and standard deviations (SD) of positive samples, and the rate of reinfection in previously seropositive children (defined as an increase of > 20 EIA units in sequential samples) are shown.

y, year of age; Seropositive defined as antibody level of > 9.5 EIA units in the sample; n, number of positive samples; GMAL, geometric mean antibody level; SD, geometric standard deviation; CI 95%, 95% confidence interval.

A subset of seropositive 1-year-old or 2-years-old children showed a diagnostic increase in HCoV S1-specific antibody levels in subsequent samples at 2 or 3 years of age, respectively, indicating a likely reinfection or re-exposure (below referred to as reinfection). Altogether 31% (27/88) of the children seropositive for any seasonal HCoV at 1 year showed a diagnostic/significant increase (more than 20 EIA units between sequential samples) in anti-S1 HCoV antibodies by the age of 2 years. A similar rate of likely reinfection for any HCoV (34%) was observed between 2 and 3 years for children who were seropositive at the age of 2 years.

### Correlation of anti-HCoV S1 and anti-HCoV N antibody levels

**Figure 2:**
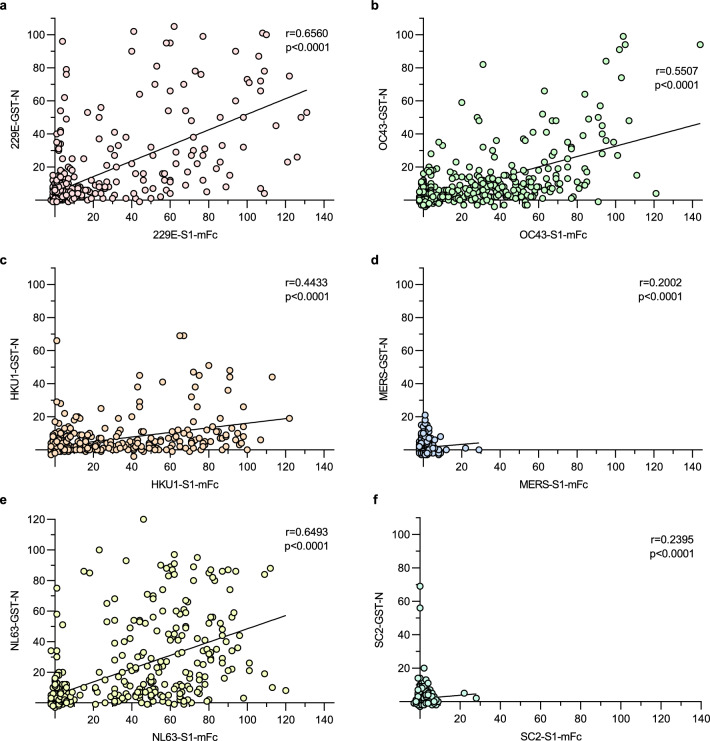
Correlation of HCoV N and S1 antibody responses. IgG antibody levels for 229E, HKU1, NL63, OC43, MERS, and SARS-CoV-2 S1 and N proteins were measured for 420 serum samples from 1 to 3-year-old children. The correlation of anti-S1 and anti-N antibody levels for each HCoV was analyzed using Spearman’s matched pairs test. The correlation coefficient (r) and two-tailed p-value for each pair is shown.

HCoV infections have been shown to induce antibodies for both HCoV S and N proteins^9,10^. We have previously analyzed anti-HCoV N IgG antibody levels for the same set of serum samples from children (n = 420)^18^ allowing us to use the existing data to analyze the correlation of N and S1 protein-specific antibody responses. We calculated the correlation coefficients for S1 and N EIA data of each HCoV (Fig. 2). The correlation coefficients of S1 and N IgG antibody levels for 229E and NL63 were relatively high (r = 0.66 and r = 0.65, respectively, p < 0.0001). The rates of correlation for anti-HKU1 S1 and N, and anti-OC43 S1 and N IgG antibodies were somewhat lower, yet significant (r = 0.44 and r = 0.55, respectively, p < 0.0001). However, the negative samples may contribute considerably to the higher rates of correlation since the removal of samples that were negative in both assays weakened the correlation of all assay pairs (Supplementary Fig. 3).

### COVID-19 vaccination has no effect on HCoV S1 IgG antibody levels in adults

**Figure 3:**
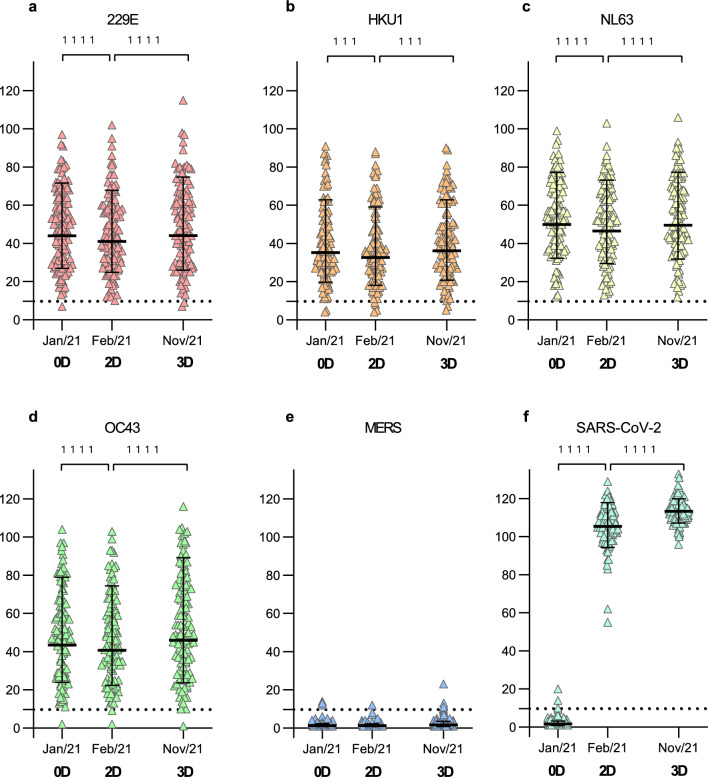
HCoV S1-specific antibody responses of COVID19 mRNA vaccinated health care workers (HCWs). (**a**–**f**) HCoV S1-specific IgG antibody levels were measured with EIA from 339 serum specimens collected before vaccination (0D, Jan/21), and three weeks after second (2D, Feb/21) and third vaccination (3D, Nov/21) from 113 HCWs who received two doses of BNT162b2 with a three-week interval in December 2020 to February 2021 and a third dose of BNT162b2 or mRNA-1273 in September to December 2021. Geometric means and geometric standard deviations of antibody levels are shown. Differences between the groups were analyzed with Wilcoxon matched pairs test. Dashed line indicates the cutoff value for seropositivity. Two-tailed p-values < 0.05 were considered statistically significant. P-values 1111 < 0.0001 and 111 (**b**) from left to right are 0.0007 and 0.0008.

To investigate whether COVID-19 vaccination induces the production of HCoV S1 cross-reactive antibodies, we analyzed sera of COVID-19 vaccinees with HCoV S1 protein-specific EIAs. Sera were collected from 113 HCWs (25–65 years old, Table 1) before vaccination with BNT162b2 (0D; August 2020 to January 2021), three weeks after the second BNT162b2 dose (2D; January to March 2021), and three weeks after the third vaccination dose with BNT162b2 or mRNA-1273 (3D; October 2021 to January 2022). Geometric mean IgG antibody levels (GMALs) for the seasonal HCoV S1 proteins decreased from 0 to 2D but increased from 2 to 3D (44 EIA units at 0D vs. 41 EIA units at 2D vs. 44 EIA units at 3D for 229E, both p < 0.0001; 35 vs. 33 vs. 36 for HKU1, p = 0.0007 and p = 0.0008; 50 vs. 46 vs. 50 for NL63, p < 0.0001; 43 vs. 41 vs. 46, for OC43 p < 0.0001; Fig. 3). GMALs for MERS S1 remained practically negative while two doses of BNT162b2 increased the SARS-CoV-2 S1 GMALs from negative to high levels (GMAL of 1.6 EIA units at 0D vs. 105 at 2D, p < 0.0001). The third mRNA vaccine dose (BNT162b2 or mRNA-1273) further boosted the SARS-CoV-2 antibody levels (GMAL of 105 EIA units at 2D vs. 113 at 3D, p < 0.0001).

Comparison of HCoV S1-specific antibody responses between genders showed similar antibody levels at each time point with the exception of anti-NL63 S1 antibodies, which were slightly, although not significantly higher in female than in male HCWs (GMAL of 37 EIA units for male vs 51 for female at 0D, 33 vs 48 at 2D, and 43 vs. 50 at 3D; Supplementary Fig. 4). Unequal number of the HCWs in the genders (9 males and 104 females) limited the strength of the comparisons between the groups.

### Prevalence of HCoV infections in 2020–2021

**Figure 4:**
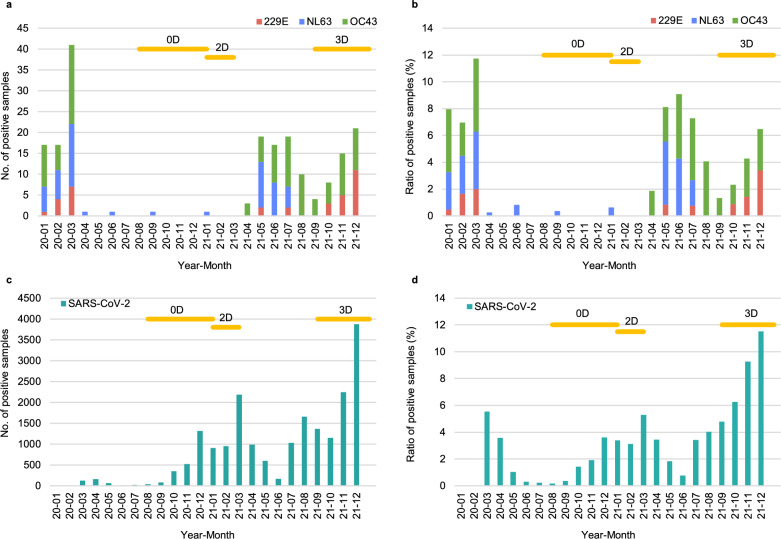
Monthly prevalence of HCoV RT-qPCR-positive samples in Southwest Finland hospital district in 2020–2021. Multiplex RT-qPCR test (with Allplex Respiratory panel 3, Seegene) including the detection of 229E, NL63, and OC43, or an in-house RT-qPCR for SARS-CoV-2 was used to analyze respiratory samples at the Clinical microbiology unit of Turku University Hospital in 2020 to 2021. Monthly numbers of positive samples for 229E, NL63, OC43 (**a**), SARS-CoV-2 (**c**) as well as the ratio of positive samples of tested samples (**b** and **d**) are presented. Horizontal orange lines indicate the timelines for serum specimens collected before (0D), three weeks after two doses (2D) and three weeks after the third (3D) mRNA vaccine dose. The number of tested samples for multiplex RT-qPCR varied from 104 to 377 (228 on average) per month and for SARS-CoV-2 RT-qPCR 2280 to 41,300 (22,983 on average) per month.

To investigate how the circulation of different HCoVs associated with the changes in HCoV antibody levels in the study population, we analyzed the monthly number and prevalence of HCoV PCR-positive samples in hospitalized patients in Southwest Finland health district in 2020–2021 (Fig. 4). The multiplex RT-qPCR with Allplex Respiratory Panel 3 (Seegene Inc.), which detected 229E, NL63, and OC43 (but not HKU1) RNA, showed the circulation of these viruses in early 2020 (January–March) with a peak of 12% of positive HCoV samples in March 2020 (7 229E-positive, 15 NL63-positive, and 19 OC43-positive out of 349 tested samples). This was followed by 12 months (April 2020 to March 2021) of very low number of positive samples (only 4 NL63-positive samples out of 2356 tested samples). Starting from April 2021 the circulation of OC43 was observed monthly until the end of the study period. NL63 was detected from May to July 2021 but no circulation was seen later in the year. 229E was detected sporadically after May 2021 and occasionally between October and December 2021. Detection of SARS-CoV-2 with laboratory developed RT-qPCR showed higher number of positive cases starting from autumn 2020 with peaks in March and December 2021.

### Congruence of seasonal HCoV occurrence and S1 IgG antibody levels

**Figure 5:**
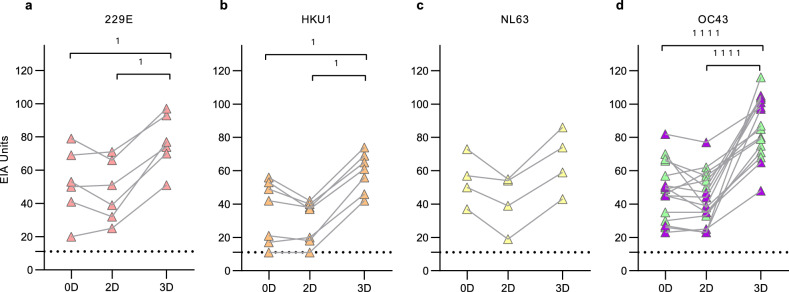
Increases in HCoV S1 binding IgG antibody levels indicate reinfection. HCWs received two doses of BNT162b2 and a third dose of BNT162b2 or mRNA-1273 in 2020–2021. Serum samples were collected before vaccination (0D), 3 weeks after the second (2D), and 3 weeks after the third dose (3D) and analyzed for 229E (**a**), HKU1 (**b**), NL63 (**c**), and OC43 (**d**) S1 binding IgG antibodies with EIA. An increase of > 20 EIA units between sequential samples was considered as an indication for reinfection or re-exposure. Sequential serum samples of different individuals are connected with lines. In figure (**d**) purple-marking indicates the antibody levels of individuals who have an increase in antibodies for both OC43 S1 and HKU1 S1 and green-marking those with an increase only in OC43 S1 binding antibodies. Statistical differences between the time points were analyzed using Wilcoxon matched pairs test. Two-tailed p-values < 0.05 were considered statistically significant. P-values from left to right marked with 1 (panels a and b) are 0.031, 0.031, 0.016, and 0.016. 1111 < 0.0001.

The absence of serologically observable seasonal HCoV reinfections (defined as > 20 EIA unit increase in antibody levels of sequential samples) between 0 and 2D (Fig. 3a–d) correlated with the low number of seasonal HCoV-positive samples in Autumn 2020 to March 2021 in Southwest Finland health district. The increases in seasonal HCoV S1 IgG antibody levels between the second and third vaccine doses (8 months apart) on the other hand suggested potential circulation of HCoVs and reinfection amongst HCWs (Fig. 5). The PCR-data on the rates of 229E, NL63, and OC43 detections from May 2021 onwards matched well with the serological changes among some of the HCWs on the same period. Diagnostic rises in antibody levels from 2 to 3D indicated a reinfection rate of 5.3% (6/113) for 229E, 6.2% (7/113) for HKU1, 3.5% (4/113) for NL63, and 14.2% (16/113) for OC43. Interestingly, each participant (n = 7) showing an increase in HKU1 S1 antibodies had also an increase in OC43 S1 antibodies (Fig. 5).

### Correlation of HCoV S1-binding antibody levels

**Figure 6:**
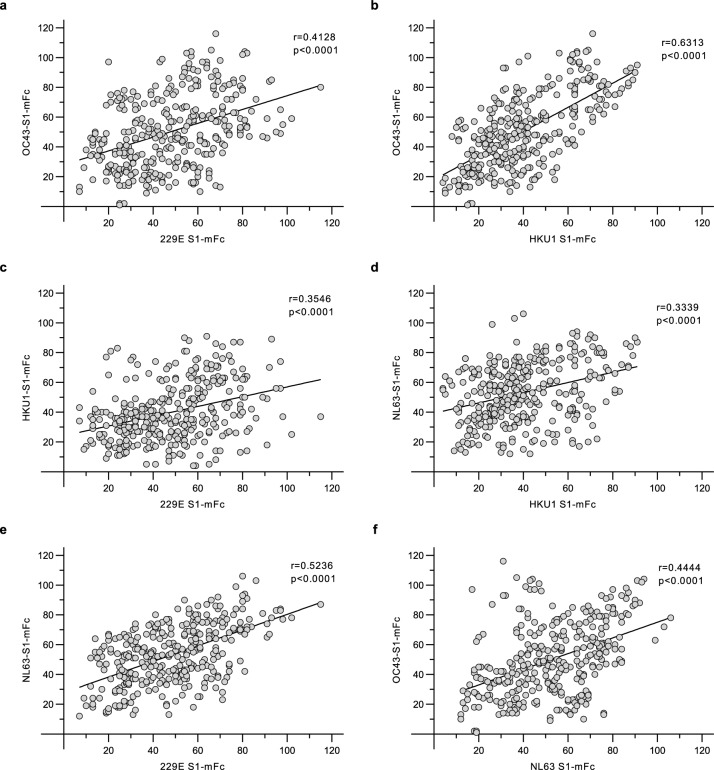
Correlation of seasonal HCoV S1 binding antibody responses. Pairwise correlation of 229E, HKU1, NL63, and OC43 S1 binding IgG antibody levels were analyzed from the data of 113 HCWs (n = 339 serum samples) with Spearman’s matched pairs test. The correlation coefficient (r) and two-tailed p-value for each pair is shown.

To evaluate the presence of homologous and potentially cross-reactive anti-S1 antibodies, we used the data from 339 serum samples of COVID-19 vaccinated HCWs to analyze the correlation of seasonal HCoV S1-binding IgG antibodies. The highest rates of correlation were observed for anti-229E and anti-NL63 S1 antibodies (r = 0.524, p < 0.0001), and anti-HKU1 and anti-OC43 S1 antibodies (r = 0.631, p < 0.0001) but also other assay pairs showed moderate correlation coefficients (r > 0.334, p < 0.0001 for other anti-HCoV S1 antibody level pairs) (Fig. 6).

In children (420 serum samples, Supplementary Fig. 5), a moderate correlation was observed for anti-HKU1 S1 and anti-OC43 S1 binding antibodies (r = 0.589, p < 0.0001) and for anti-229E S1 and anti-NL63 S1 binding antibodies (r = 0.512, p < 0.0001) while the correlation coefficient values of other anti-HCoV S1 pairs was lower (r < 0.26). Pairwise comparison of S1 amino acid sequences (Supplementary Table 1) showed that the pairs of 229E and NL63, as well as HKU1 and OC43 shared more identical amino acids (50% and 59%, respectively) in comparison to other sequence pairs (10–22%), which may contribute to sequence identity-related immunological correlation.

The correlation coefficient values for anti-SARS-CoV-2 S1 antibodies and other anti-HCoV S1 antibodies after COVID-19-vaccination were low at 2D and 3D time points (Supplementary Fig. 6). Despite the low correlation coefficient values, likely due to mainly seronegative specimens, the correlation of anti-SARS-CoV-2 S1 antibodies was statistically significant (p < 0.05) with anti-OC43 antibodies at 2D (r = 0.3033, p = 0.0011), and with anti-MERS antibodies both at 2D and 3D (r = 0.2462, p = 0.0086 and r = 0.2098, p = 0.0257, respectively).

### Homologous and cross-reactive S1 antibodies in immunized animals

The data from HCoV reinfections in HCWs as well as the correlations between anti-S1 antibody levels suggested potential cross-reactivity between HCoV S1-specific antibodies. To validate further the antigens chosen for EIA, we immunized rabbits and guinea pigs with the HCoV S1 proteins without mFc-fusion (except for OC43 for which S1 with mFc was used due to unsuccessful production of OC43 S1 without mFc, Supplementary Fig. 1). Each animal was immunized with one antigen. Antigens without mFc-fusion were used in immunization to avoid formation of antibodies against the mFc domain. The specificity of immune sera was examined by immunofluorescence (IF) assay for homologous and cross-reactive antibodies using 229E, OC43, and NL63 virus-infected cells (Huh7 or LLC-MK2). Virus-infected cells were detected with high IF titers (1:1000 to 1:5000) by the corresponding anti-S1 sera of immunized rabbits and guinea pigs (Table 3, Supplementary Fig. 7). In addition to homologous reactivity of sera, cross-reactivity was also observed. Cells infected with 229E were recognized by anti-HKU1 S1 rabbit serum (titer 50) and by both rabbit and guinea pig anti-NL63 S1 sera (titer 200; Table 3, Supplementary Fig. 8). OC43-infected cells were recognized by rabbit and guinea pig anti-HKU1 S1 sera (titer 200, Supplementary Fig. 9) and NL63-infected cells were recognized by rabbit anti-OC43 S1-mFc sera (titer 200, Supplementary Fig. 10).

**Table 3:** Cross-reactive and homologous titers of serum from HCoV S1 immunized rabbit and guinea pig.

Serum	229E IF titer	NL63 IF titer	OC43 IF titer
Rb anti-229E S1	**5000**	< 50	< 50
Gp anti-229E S1	**1000**	< 50	< 50
Rb anti-HKU1 S1	< 50	< 50	**200**
Gp anti-HKU1 S1	**50**	< 50	**200**
Rb anti-NL63 S1	**200**	**5000**	< 50
Rb anti-NL63 S1	**200**	**5000**	< 50
Rb anti-OC43 S1	< 50	**200**	**5000**
Gp anti-OC43 S1	< 50	< 50	**5000**
Rb anti-SARS-CoV-2 S1	< 50	< 50	< 50
Gp anti-SARS-CoV-2 S1	< 50	< 50	< 50

Titers for sera of one rabbit and one guinea pig immunized with three doses of each of the indicated HCoV S1 antigens were determined using immunofluorescence assay. Huh7 cells were infected with 229E or OC43 virus and LLC-MK2 cells with NL63 virus and labelled with a dilution series (1:50 to 1:25,000) of sera against each HCoV S1. Serum titer > 1:50 was considered positive. Of note, infectious HKU1 is globally not available.

Rb, rabbit; Gp, guinea pig; S1, spike subunit 1; IF titer, immunofluorescence titer; < 50, IF titer lower than 1:50.

Significant values are in bold.

## Discussion

In this study we investigated the seroprevalence of HCoV spike-specific antibodies by analyzing anti-HCoV IgG antibodies in sequential serum samples collected from 140 children between 1 and 3 years of age and from BNT162b2-vaccinated HCWs in three time points: before the vaccination and after two and three vaccine doses. Spike subunits 1 (S1) were chosen as antigens for the EIA since they are genetically more distant from each other compared to the whole spike proteins or the S2 subunits. The spike protein is the main structural protein on the coronavirus surface, and it is responsible for the binding and fusion of the virus with the target cell, and due to its immunogenicity, it is a good antigen for serological studies. Dimeric S1-mFc fusion proteins were chosen as antigens for serological assays due to their enhanced stability and reliable functionality in EIA^10^.

We detected somewhat higher seroprevalence rates for anti-HCoV S1 antibodies as compared to the seroprevalence rates of anti-HCoV N antibodies in the same child population^18^. This observation is well in line with previous studies showing a high seroprevalence for binding antibodies against the whole HCoV S proteins^19^ and somewhat more variation in seroprevalence for HCoV N antibodies^3,12,13^. Also, based on the cumulative seroprevalence, the waning of anti-S1 antibodies as shown by the present study and previous publications was slower compared to the waning of anti-N antibodies^8,18^. Despite the relatively high correlation coefficient values for anti-N and anti-S1 antibodies for four seasonal HCoVs, our data shows that in some individuals HCoV infections induce the production of IgG antibodies only against one of these antigens, an observation similar to the one seen in SARS-CoV-2 infections^9,10^.

The PCR data suggested a low rate of circulation of HCoVs from April 2020 to March 2021, which is well in line with the reports on limited circulation of endemic seasonal respiratory viruses^20,21^ due to “lockdown” of the society and other measures used to prevent the spread of the COVID19 pandemic. The data also showed that two doses of BNT162b2 did not increase antibody levels against other HCoV S1 proteins indicating practically a complete lack of immunological cross-reactivity. The gradual release of COVID-19 restrictions in the spring of 2021 in Finland^22^ resulted in the circulation of seasonal HCoVs followed by an increase in anti-HCoV S1 antibody levels in the autumn 2021. Furthermore, the reinfection events of OC43, seen as a diagnostic increase in the anti-OC43 S1 antibodies between 2 and 3D serum specimens, matched well with the circulation of OC43 during the year 2021. In epidemiological studies the occurrence of HKU1 has been lower than that of other seasonal HCoVs^23,24^. Thus, the high seroprevalence for HKU1 in this study may be due to cross-reactive antibodies induced by OC43 infections. In our analysis all individuals showing an increase in anti-HKU1 antibodies also had an increase in anti-OC43 antibodies indicating that immunological cross-reactivity between OC43 and HKU1 may affect the anti-HKU1 antibody positivity rate. Similar immunological cross-reactivity between OC43 and HKU1 spike protein was suggested by our analyses of rabbit and guinea pig immune sera.

Data from immunized animals, antibody level correlation coefficient values and HCW serum analyses with indications of seasonal HCoV reinfections suggest significant cross-reactivity between anti-HKU1 and anti-OC43 S1 antibodies and anti-229E and anti-NL63 S1 antibodies, respectively. Sequence identities between the above HCoV S1 protein pairs is higher than those of other S1 protein pairs and similar cross-reactivity of antibodies exist with the corresponding anti-N antibody pairs^18,23,25^. Immunizations with OC43 S1 were done with mFc-fusion of the S1 and the effect of mFc-specific antibodies into cross-reactivity cannot be completely ruled out, although our data indicates no such reactivity. Immunological cross-reactivity between HKU1 and OC43 or 229E and NL63 may also provide cross-protection even though we have no experimental evidence for that. The level of cross-reactive antibodies as well as the cross-reactive HCoV pairs would likely be greater if the full-length spike protein or the S2 subunit were used as the antigens due to higher sequence similarities in these protein sequences. For example, OC43 and HKU1 S2-specific antibody responses have been described to correlate with antibody responses induced by SARS-CoV-2 infection^26,27^. However, since our prevaccination sera were devoid of binding activity to SARS-CoV-2 S1 protein and COVID-19 vaccination did not induce any increase in seasonal HCoV S1-specific antibody responses, we consider it quite unlikely that there is humoral cross-protection induced by seasonal HCoV infections against SARS-CoV-2 and vice versa.

Our data shows that the number of children with HCoV S1-binding IgG antibodies increases by age, reaching 40–80% by the age of three years. In adults these values are nearly 100% against all the seasonal HCoVs indicating a high rate of circulation of 229E, NL63 and OC43 viruses. Twelve-month follow-up of vaccinated adults showed that increased circulation of HCoVs matched relatively well with changes in anti-HCoV antibody levels. These observations provide further information on the characteristics of humoral immune responses of coronavirus infections which appear to be extremely common respiratory pathogens also among the adults.

## Materials and methods

### Study participants

Infant participants were part of the STEPS study^28^, a prospective observational birth-cohort study in the Southwest Finland health district. Serum samples were collected at 13.7 (1y), 25.3 (2y) and 37.5 (3y) months of age from 140 children (53% male and 47% female; n = 420 samples) during visits to a study clinic in 2009 – 2013, and the same serum specimens have been used in our previous study concentrating on anti-HCoV nucleoprotein antibodies^18^. The Ethics Committee of the Southwest Finland health district approved the study (decision no. 16/180/2008). Parents of participating children gave their written, informed consent.

Healthcare workers (HCWs, n = 113, 92% female and 8% male) aged 25–65 years (average 43 years) had obtained two doses of BNT162b2 with a three-week (21 days for each participant) dosing interval between the first two doses in December 2020–February 2021 and a BNT162b2 or an mRNA-1273 as the third vaccine 9 months (7.7–10.3 months post the 2nd dose, 8.5 months as an average) later in September–December 2021. The participants were randomly selected for this study from a larger cohort^29^. Serum samples from the vaccinees were collected 0–129 days (average 22.7 days) before the vaccination (0D) in August 2020 to January 2021, 3 weeks (17–49 days, average 24.2 days) after the second dose (2D) February to March 2021, and 3 weeks (15–44 days, average 24.4 days) after the third dose (3D) in September to December 2021. Ethical permission for this study was obtained from the Southwest Finland health district (ETMK 19/1801/2020, EudraCT 2021-004419-14). All participants signed a written informed consent at the enrollment for the study.

### Recombinant HCoV spike protein S1 antigen production

Codon-optimized genes encoding the spike protein subunit 1 (S1) of 229E (GenBank accession KY621348.1, residues 1–565), HKU1 (KY674943.1, residues 1–750), NL63 (KY554967.1, residues 1–717), OC43 (MN306053.1, 1–760), SARS-CoV-2 (NC_045512.2, residues 16–541), and MERS (JX869059.2, residues 1–747) were obtained from GeneUniversal. The HCoV S1 genes were cloned into a vector with a C-terminal mouse IgG2a Fc part (mFc) tagged with polyhistidine (His) tail, and a vector with only the C-terminal His tail. HCoV S1-mFc-His (referred to as HCoV S1-mFc) proteins, the EIA antigens, and the HCoV S1-His (HCoV S1 without mFc) proteins were produced in HEK-293F cells, and purified as described previously^10^.

### HCoV anti-S1 and anti-N enzyme immunoassays

The presence of HCoV S1 binding IgG antibodies (anti-HCoV-S1) in the serum samples was analyzed with an enzyme immunoassay (EIA) using HCoV-S1-mFc proteins. EIA was conducted with 1:300 dilution of serum samples and the quantification of antigen-binding IgG was done with measurement of absorbance at wavelength of 450 nm as described previously^10,18^. The protocol was adjusted slightly to optimize the assay for the detection of lower IgG concentrations without saturation in the higher IgG concentrations: coating concentration for S1-mFc proteins was 2.0 µg/ml and the dilution of horseradish peroxidase (HRP) conjugated anti-human antibodies 1:6000 (Dako A/S).

The optical density (OD) value of each sample was converted into EIA units by comparing it to the OD values of positive (marked as 100 EIA units) and negative (marked as 0) control sample pools measured by SARS-CoV-2 S1 EIA. To allow the calculation of geometric means the samples with an EIA unit value lower than 1 were marked as 1. The cutoff value was defined as the mean of anti-SARS-CoV-2 S1 EIA results from the negative child serum samples (n = 417, one child showed detectable antibodies and therefore it was excluded in the calculations) plus five standard deviations (SD). The inter-assay variation was controlled with an additional control sample pool and observed to remain < 10% except for anti-MERS S1 for which no positive control was available. Based on the limited inter-assay variation we considered a > 20 EIA unit increase in antibody levels to indicate a reinfection.

### Immunization of rabbits and guinea pigs

A total of five rabbits (Hsdlf:NZW, female, 13–14 weeks of age) and five guinea pigs (HsdDhl:DH, female, 12–14 weeks of age) obtained from Envigo (The Netherlands), were acclimatized for 2 weeks before being immunized. A rabbit and a guinea pig were immunized with three 50 µg-doses of either 229E S1-His, HKU1 S1-His, NL63 S1-His, or OC43 S1-mFc-His protein or four 50 µg-doses of SARS-CoV-2 S1-His protein. Each animal was immunized with one antigen. Immunizations were carried out with a 2-week dosing interval and the proteins were administered with adjuvant system 03 (AS03, GlaxoSmithKline) intramuscularly (rabbits) or subcutaneously (guinea pigs).

Both rabbits and guinea pigs were sampled for serum before the first immunization (0-sample) and at the end of the immunization 7–10 days after the last booster dose. The rabbits were additionally sampled at the time of each immunization. The animals were anesthetized with isoflurane (guinea pigs) or with subcutaneous injection of medetomidine and ketamine injection (rabbits) and euthanized by cardiac puncture. Project Authorization Board of Finland approved the procedures and protocols (license numbers: ESAVI/16751/2020 and ESAVI/20869/2020) in accordance with the 2010/EU/63 EU Directive and Finnish national legislation (497/2013 and 564/2013) on the protection of animals used for scientific purposes.

### Indirect immunofluorescent (IF) assay with cells infected with 229E, NL63, and OC43

LLC-MK2 (rhesus monkey kidney) cells and Huh7 (human hepatoma) cells were maintained in Dulbecco’s modified Eagle’s medium (DMEM, Lonza BioWhittaker) supplemented with 10% FBS (Biowest Nuaille), 1% l-glutamine (ThermoFischer Scientific), and 1% penicillin/streptomycin (Lonza). For the infections the concentration of FBS in the medium was reduced to 2%. Infections of Huh7 cells with 229E (GenBank Accession OK662398) and OC43 (OK662397) and the following fixing and permeabilization for IF were done as described previously^18^. LLC-MK2 cells were infected with 0.1 FFU/ml of NL63 (AY567487.2, a kind gift from Prof. van der Hoek) for 48 h followed by fixing the cells with 4% paraformaldehyde and permeabilization with 0.1% Triton-X-100 in PBS. An infectious virus for HKU1 is globally not available and thus HKU1 virus infected cells could not be used in IF.

Rabbit and guinea pig sera were diluted 1:50, 1:200, 1:1000, 1:5000, and 1:25,000 into PBS supplemented with 3% bovine serum albumin (BSA). Dilution series was added onto virus- or mock-infected cells in duplicates and incubated for 1 h. For double-staining of 229E-infected, OC43-infected, and NL63-infected cells the guinea pig serum dilutions were supplemented with a 1:1000 dilution of polyclonal rabbit anti-229E N-GST, rabbit anti-OC43 N-GST, or rabbit anti-NL63 sera and the rabbit serum dilutions were supplemented with corresponding guinea pig anti-HCoV N-GST sera (Huttunen et al., unpublished data), which had been prepared in serial immunizations as described above. The antibodies were detected with 1:1000 dilution of secondary antibodies goat anti-rabbit IgG 488/564 and/or goat anti-guinea pig IgG 488 (Thermo Scientific) and 1:2500 dilution of DAPI for 1 h. Antibody-binding to infected cells was imaged on EVOS FL Auto Fluorescence Inverted Microscope (Life Technologies).

### Statistical and sequence analysis

Statistical analysis of EIA results was conducted using GraphPad Prism 8 software as the platform. Wilcoxon matched pair signed-rank test corrected with Pratt’s method was used for the analysis of pairwise differences between the groups. Spearman’s correlation test was used to analyze the correlation of results between two different HCoV S1 type specific assays. Two-tailed p-values < 0.05 were considered significant. Exact p-values are shown in the figures and marked according to their significance with stars: p-value of < 0.05 *, < 0.002 **, < 0.0002 ***, and < 0.00001 ****.

Amino acid sequences of the spike, spike subunits 1 and 2, and nucleoprotein for the HCoV isolates used in this study were aligned using ClustalW^30^ in MEGA 11^31^ platform and compared pairwise for sequence identity.

### Ethics statement

All experiments were performed in accordance with relevant guidelines and regulations. The Ethics Committee of the Southwest Finland health district approved the study (decision no. 16/180/2008). Parents of participating children gave their written, informed consent. All methods are reported in accordance with ARRIVE guidelines (https://arriveguidelines.org) for the reporting of animal experiments. Procedures and protocols concerning animal experiments were approved by the Project Authorization Board of Finland (license numbers: ESAVI/16751/2020 and ESAVI/20869/2020) in accordance with the 2010/EU/63 EU Directive and Finnish national legislation (497/2013 and 564/2013) on the protection of animals used for scientific purposes.

## Supplementary Information

Supplementary Information 1.

Supplementary Information 2.

## Data Availability

The datasets generated and/or analysed during the current study are available in the supplementary info file or in the NCBI GenBank repository (https://www.ncbi.nlm.nih.gov/genbank/) under accession numbers KY621348.1, KY674943.1, KY554967.1, MN306053.1, NC_045512.2, JX869059.2, OK662398, OK662397, and AY567487.2.
