# Supplementary material for: Coronavirus spike protein-specific antibodies indicate frequent infections and reinfections in infancy and among BNT162b2-vaccinated healthcare workers
Source: Sci Rep. 2023 May 24;13:8416. doi: 10.1038/s41598-023-35471-3 (PMC10206561; doi:10.1038/s41598-023-35471-3)
Supplement: Supplementary file 2 — Supplementary Information 2. [file 41598_2023_35471_MOESM2_ESM.pdf]

Coronavirus spike protein-specific antibodies indicate frequent infections and reinfections in infancy and among BNT162b2-vaccinated healthcare workers

Supplementary data

Supplementary Table 1. Pairwise amino acid sequence identities of human coronavirus spike protein, spike subunit 1 (S1), spike subunit 2 (S2), and nucleoprotein. Amino acid sequences for human coronaviruses encoding spike, spike subunit 1, spike subunit 2, and nucleoprotein were aligned using Clustalw and compared for pairwise identities in Mega 8 software. GenBank accession numbers for the sequences used in the comparison were KY621348.1 for 229E, KY674943.1 for HKU1, KY554967.1 for NL63, MN306053.1 for OC43, NC_045512.2 for SARS-CoV-2, and JX869059.2 for MERS.

**Spike**	length (aa)	229E	HKU1	NL63	OC43	MERS	SARS	SARS-CoV-2
229E	1353	-	23 %	63 %	23 %	23 %	24 %	24 %
HKU1	1356			22 %	65 %	30 %	29 %	28 %
NL63	1171				22 %	20 %	22 %	21 %
OC43	1356					31 %	30 %	30 %
MERS	1358						28 %	27 %
SARS	1255							78 %
SARS-CoV-2	1273							-
**S1**	length (aa)	229E	HKU1	NL63	OC43	MERS	SARS	SARS-CoV-2
229E	565	-	13 %	50 %	12 %	11 %	11 %	12 %
HKU1	750			12 %	59 %	21 %	21 %	19 %
NL63	717				11 %	10 %	12 %	10 %
OC43	760					20 %	22 %	21 %
MERS	747						18 %	17 %
SARS	676							66 %
SARS-CoV-2	682							-
**S2**	length (aa)	229E	HKU1	NL63	OC43	MERS	SARS	SARS-CoV-2
229E	606	-	35 %	75 %	34 %	34 %	34 %	35 %
HKU1	639			34 %	73 %	44 %	38 %	38 %
NL63	606				35 %	34 %	32 %	33 %
OC43	606					46 %	40 %	40 %
MERS	598						41 %	42 %
SARS	579							91 %
SARS-CoV-2	591							-
**Nucleoprotein**	length (aa)	229E	HKU1	NL63	OC43	MERS	SARS	SARS-CoV-2
229E	389	-	25 %	48 %	24 %	24 %	26 %	25 %
HKU1	441			22 %	66 %	32 %	31 %	32 %
NL63	337				22 %	24 %	25 %	24 %
OC43	448					32 %	31 %	30 %
MERS	413						49 %	50 %
SARS	422							91 %
SARS-CoV-2	419							-

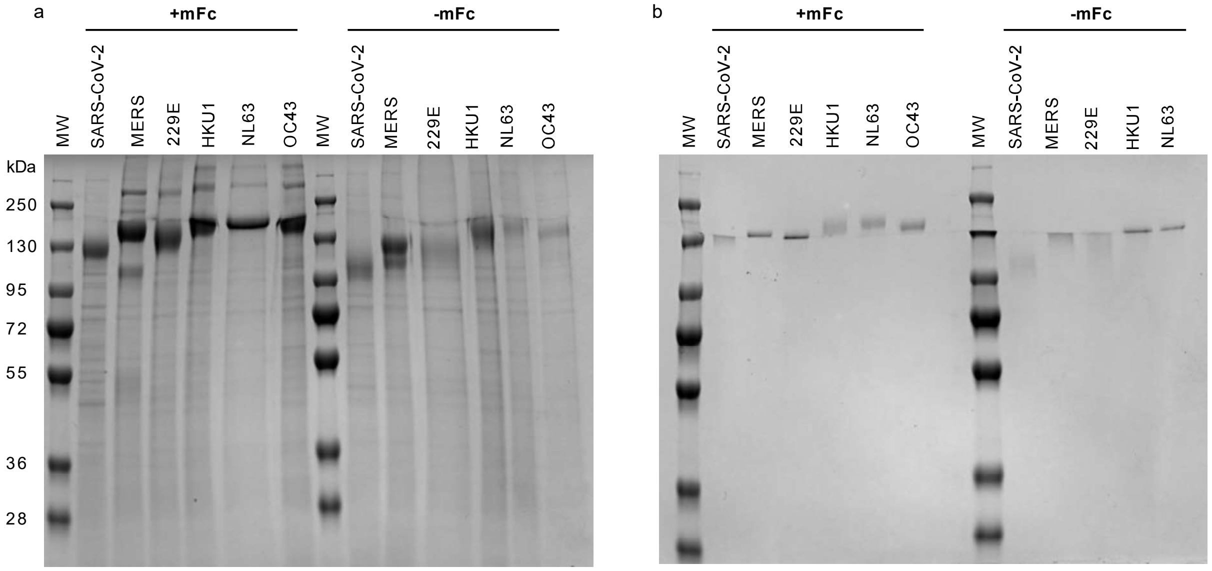

Supplementary Figure 1. Expression of recombinant HCoV S1 proteins with and without mFc-fusion. Six recombinant HCoV S1 proteins were expressed in HEK293 cells. Coomassie stained SDS-PAGE of cell suspensions (**a**) and purified proteins (**b**) of S1 proteins with mFc-fusion (+mFc) or without mFc (-mFc) of SARS-CoV-2, MERS, 229E, HKU1, NL63, and OC43 are presented. MW is the molecular weight marker.

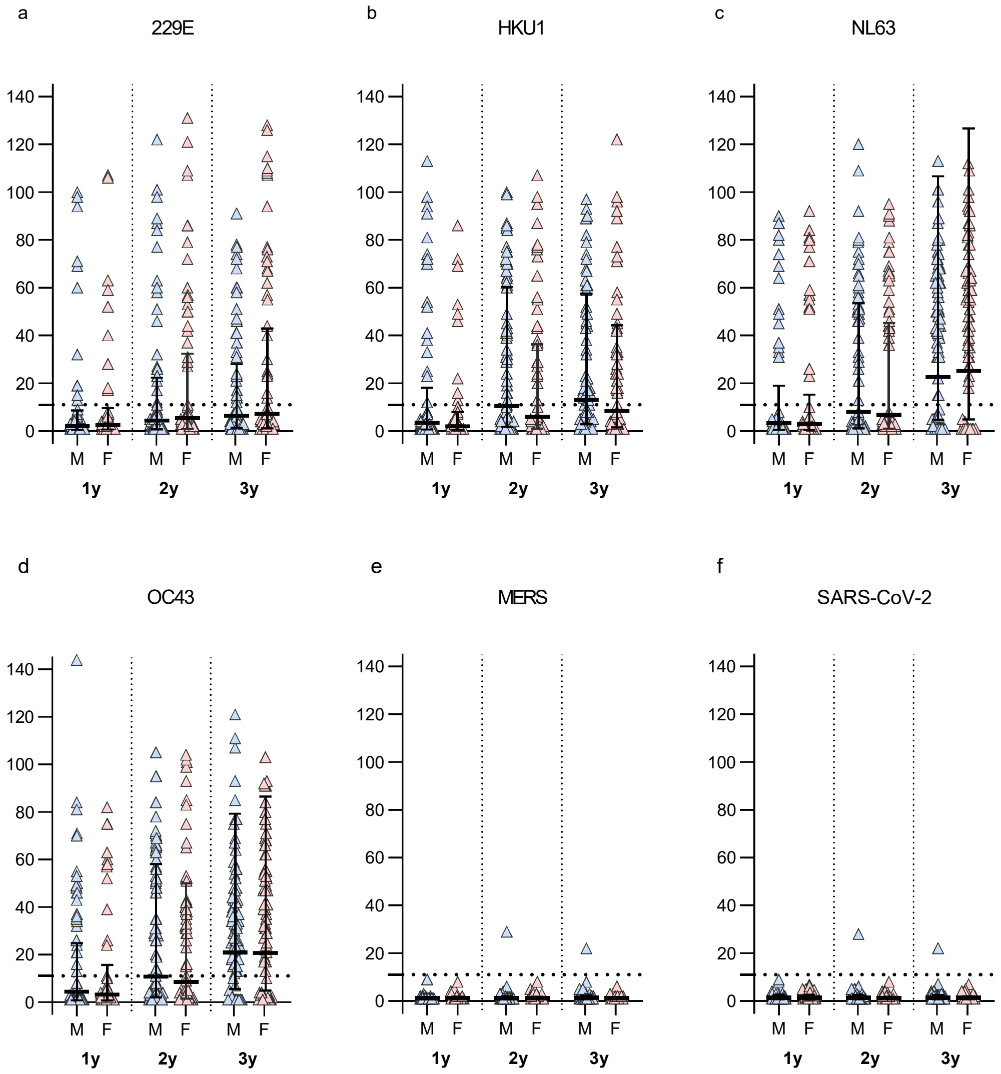

Supplementary Figure 2. Comparison of HCoV S1-specific antibody responses of 1-3 years old children based on gender. **a-f** EIA was used to measure HCoV S1-specific IgG antibody levels in serum of 74 male (M) and 66 female (F) children at the age of 1, 2, or 3 years. Geometric means and geometric standard deviations of antibody levels are shown. Dashed line indicates the cutoff value for seropositivity. Differences in the antibody levels at each time point were compared between the genders with non-paired Kruskal-Wallis test. None of the differences between gender groups had a statistically significant p-value <0.05.

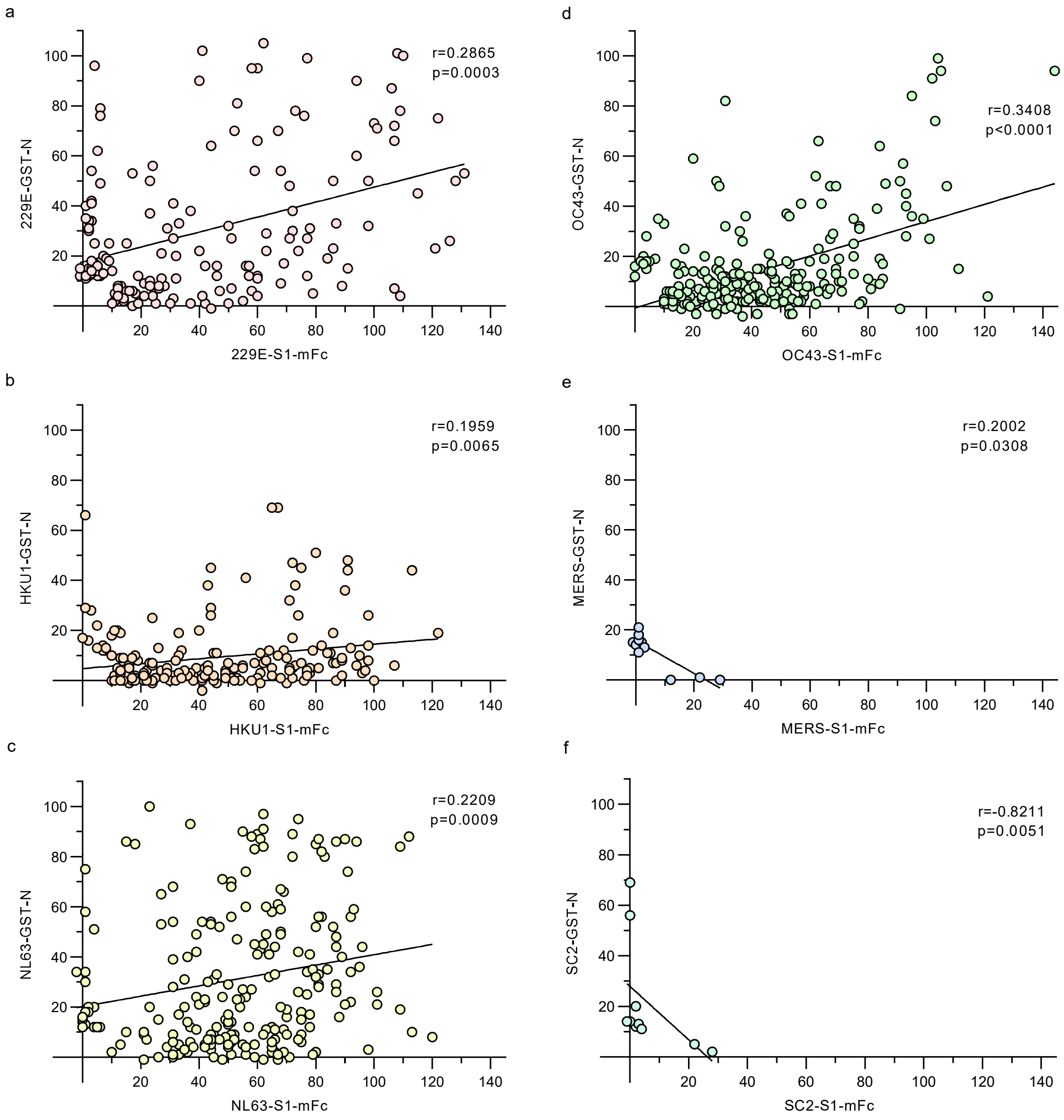

Supplementary Figure 3. Correlation of HCoV N and S1 antibody responses in children who were antibody-positive for one or both of the antigens. IgG antibody levels for 229E, HKU1, NL63, OC43, MERS, and SARS-CoV-2 S1 and N were measured for 420 serum samples from 1 to 3 years-old children. The correlation of S1 and N antibody levels for each HCoV was analyzed using Spearman's matched pairs test. The correlation coefficient (r) and two-tailed p-value for each pair is shown.

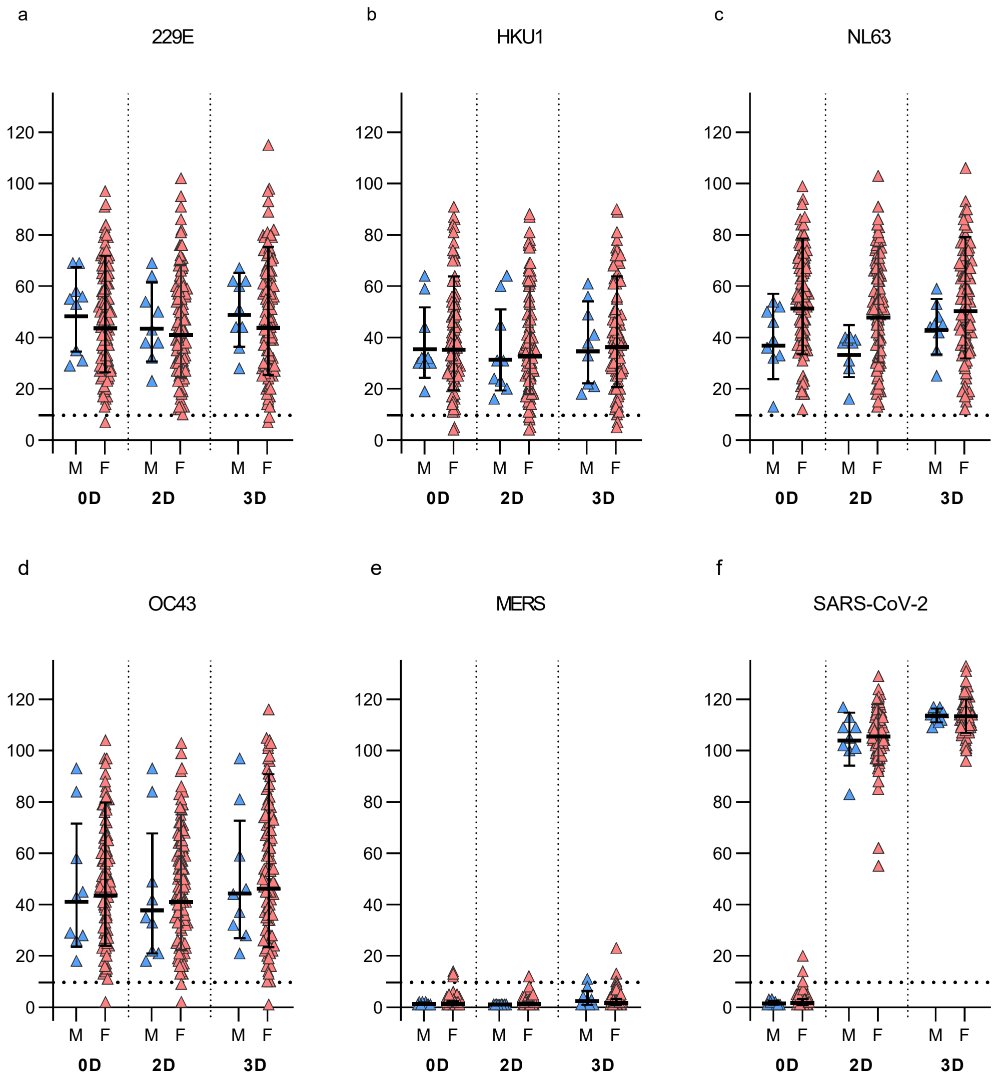

Supplementary Figure 4. Comparison of S1-specific antibody responses of COVID19 mRNA vaccinated health care workers (HCWs) between genders. **a-f** HCoV S1-specific IgG antibody levels were measured with EIA from serum collected before vaccination (0D), and three weeks after second (2D) and third vaccination (3D) of 9 male (M) and 104 female (F) HCWs. Geometric means and geometric standard deviations of antibody levels are shown. Dashed line indicates the cutoff value for seropositivity. Differences in the antibody levels at each time point were compared between the genders with non-paired Kruskal-Wallis test. None of the differences between gender groups had a statistically significant p-value <0.05.

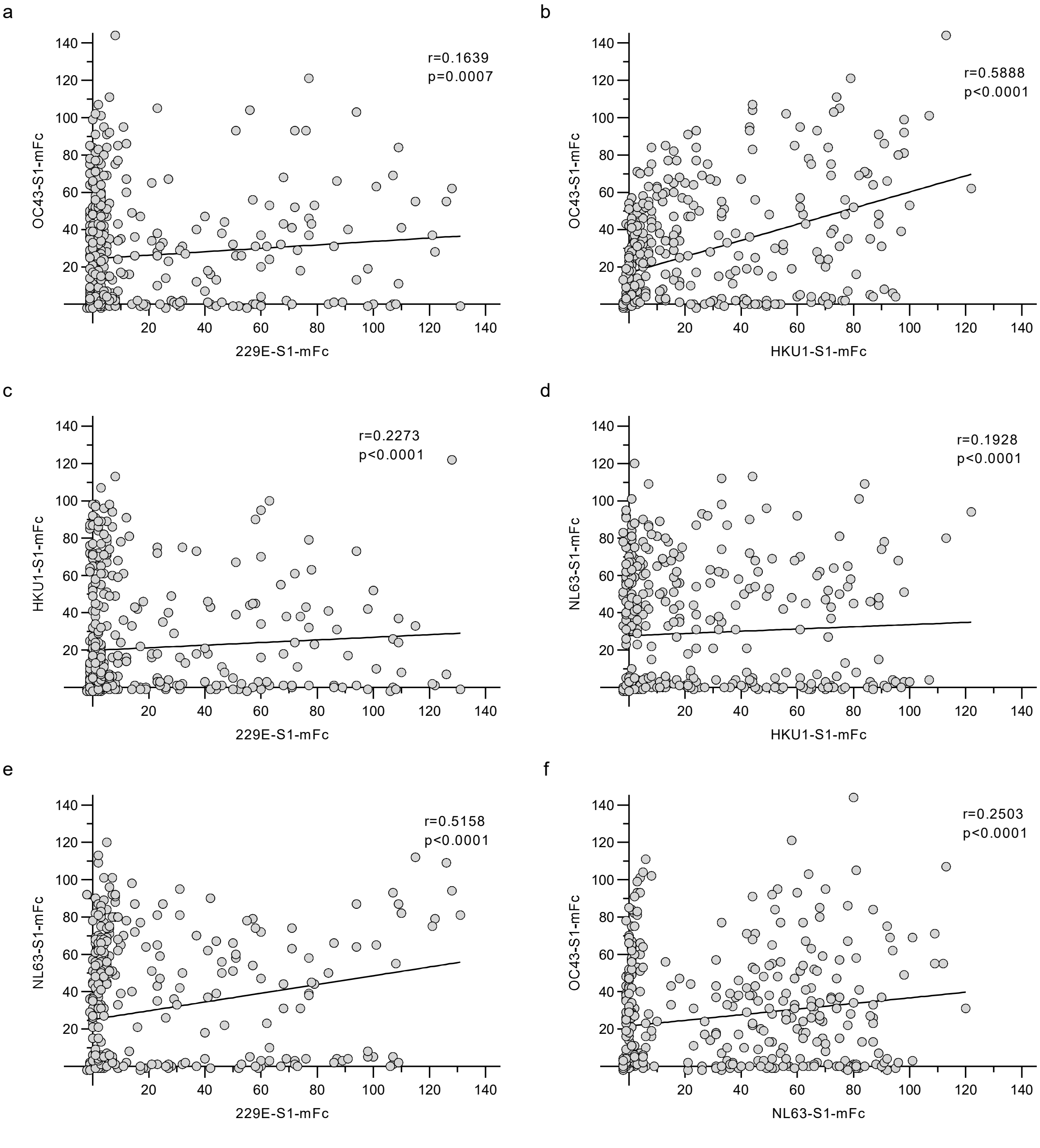

Supplementary Figure 5. Correlation of seasonal HCoV S1 antibody responses in children. Pairwise correlation of 229E, HKU1, NL63, and OC43 S1-binding IgG antibody levels were analyzed using data from 420 serum samples from children with Spearman's matched pairs test. The correlation coefficient (r) and two-tailed p-value for each pair is shown.

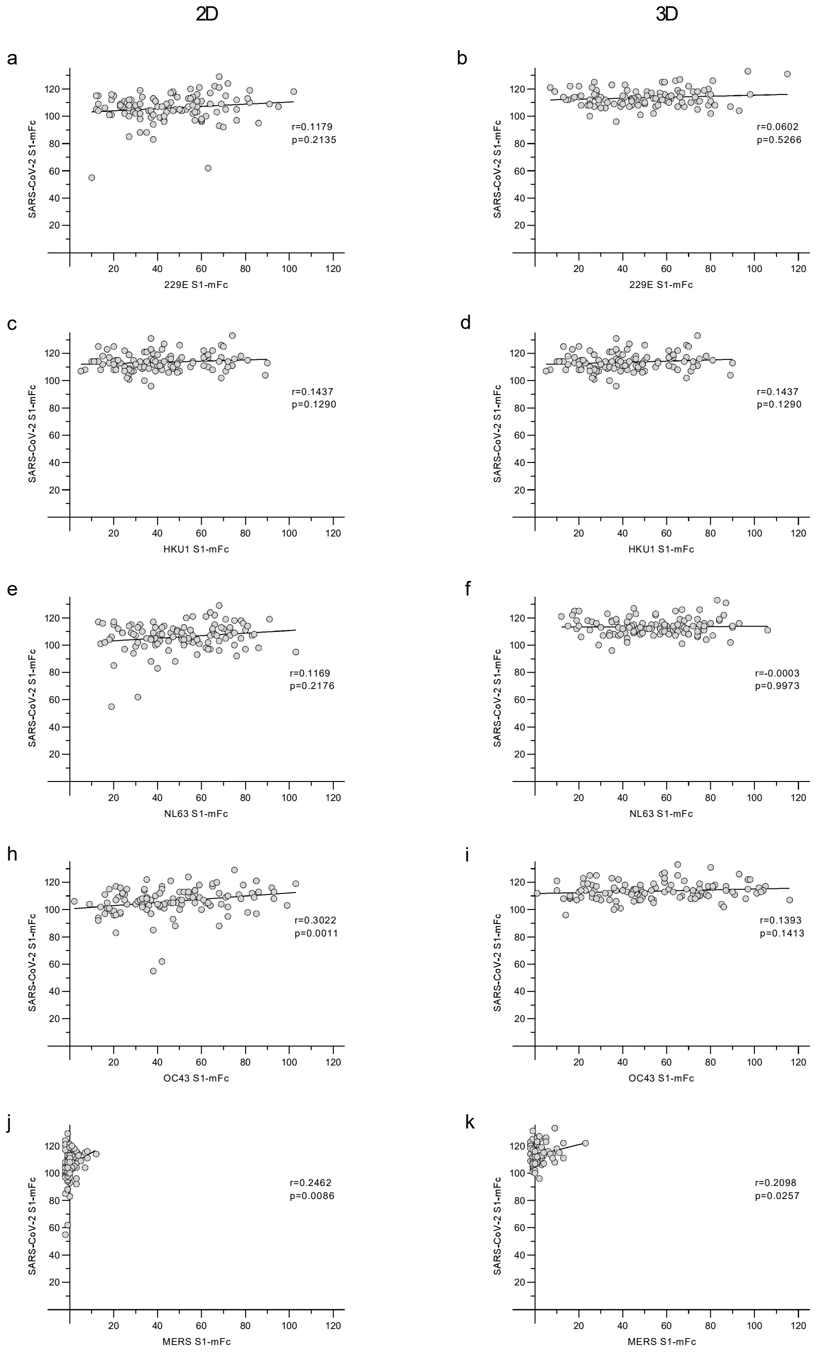

Supplementary Figure 6. Correlation of SARS-CoV-2 S1 and other HCoV S1 antibody responses. Pairwise correlation of 229E, HKU1, NL63, OC43, and MERS S1 IgG antibody levels with SARS-CoV-2 S1 IgG antibody levels were analyzed with Spearman's matched pairs test using data from 226 post-vaccination serum samples from HCWs. The correlation coefficient (r) and two-tailed p-value for each pair is shown.

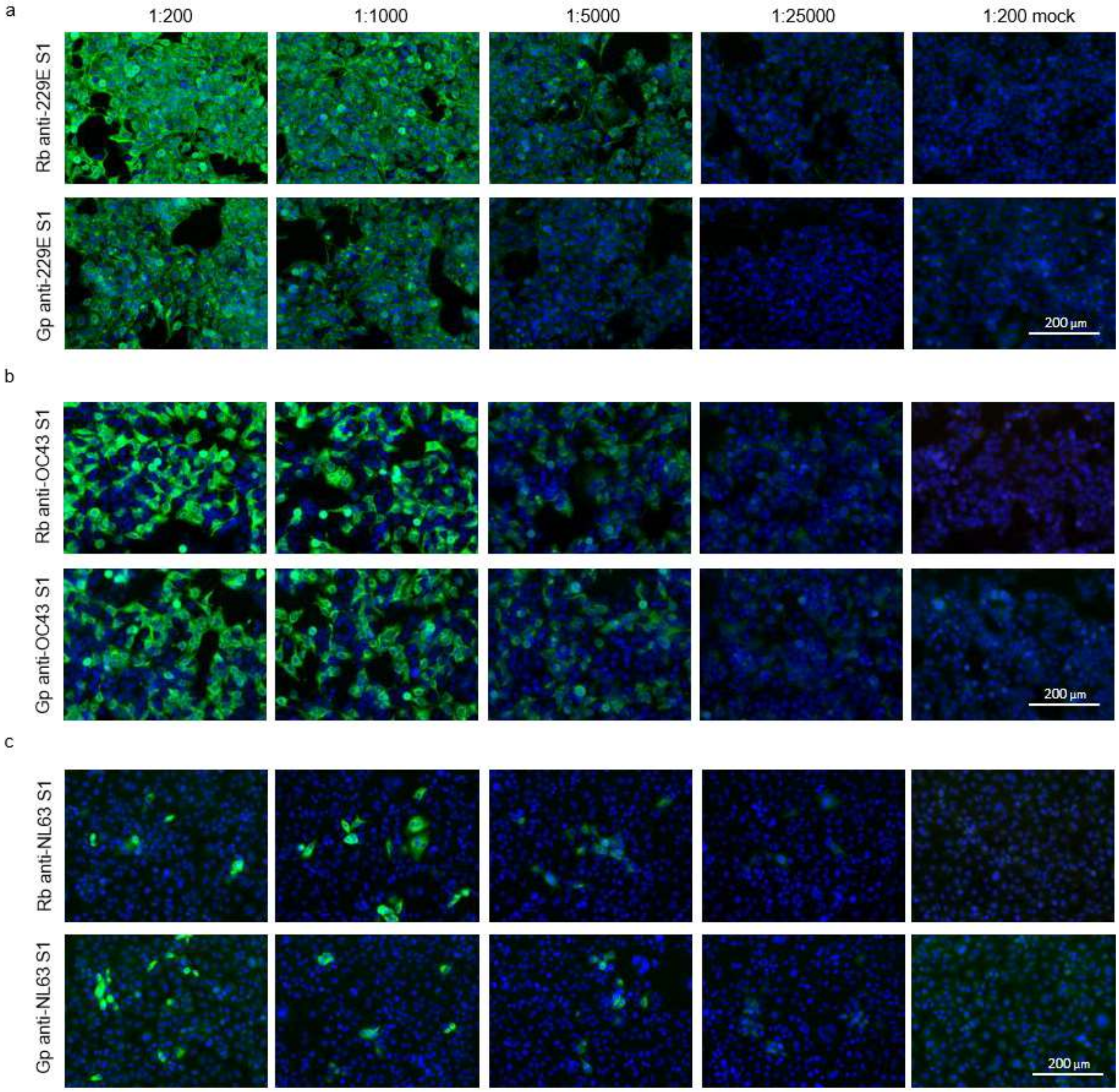

Supplementary Figure 7. Determination of immunofluorescence titers of immune sera. Huh7 cells were infected with 229E (panel a), OC43 (b) and LLC-MK2 cells with NL63 (c) viruses. Post infection (24 hours for Huh7 and 48 hours for LLC-MK2) the cells were fixed and labeled for immunofluorescence (IF) assay using serial dilutions (1:200 to 1:25000) of the corresponding anti-S1 sera. Rb, rabbit antiserum; Gp, guinea pig antiserum.

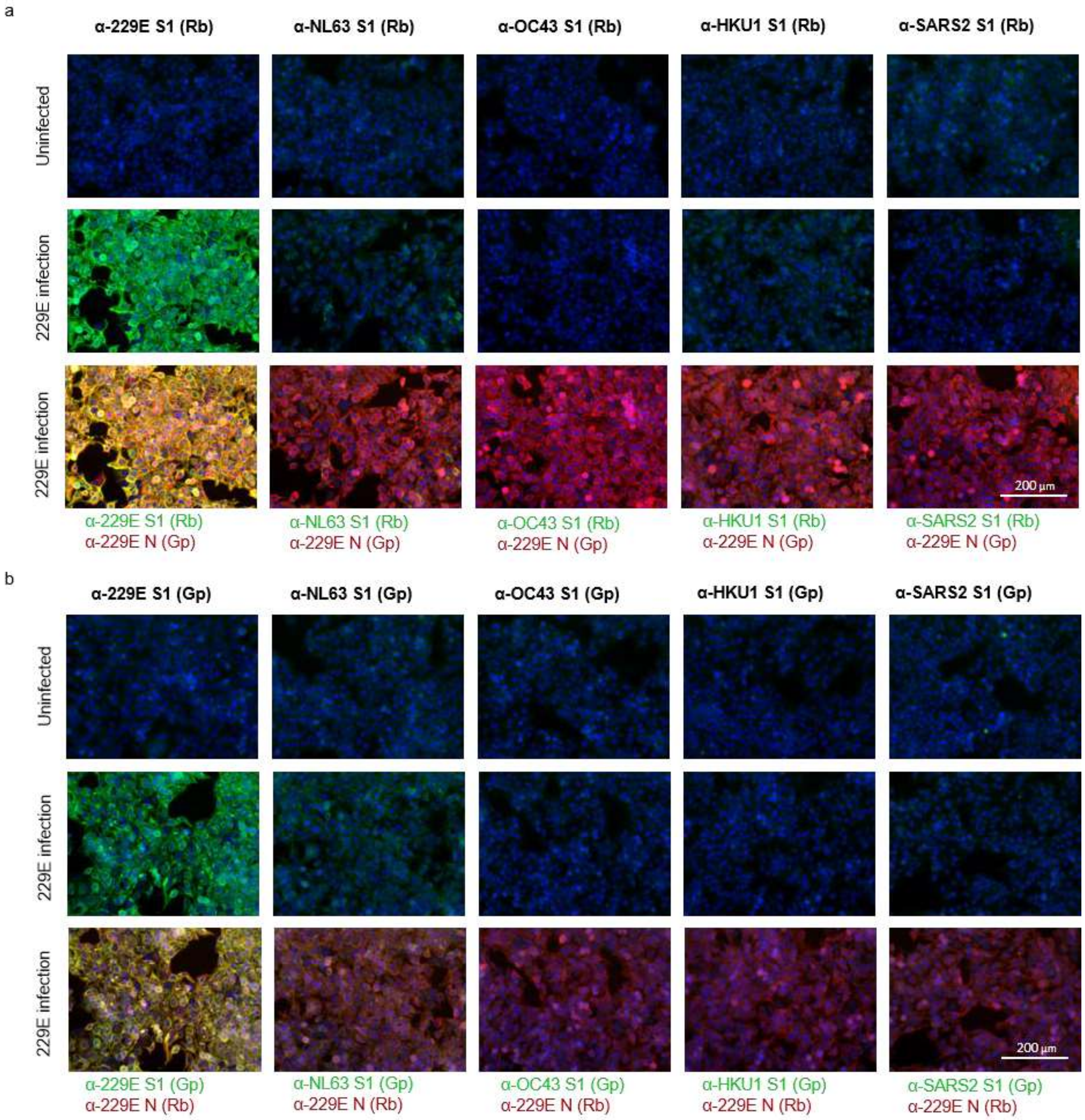

Supplementary Figure 8. Reactivity of anti-HCoV-S1 sera with 229E virus-infected cells in immunofluorescence. The titer of the reactivity of immune sera from HCoV S1protein immunized rabbits and guinea pigs was determined in immunofluorescence assay of 229E virus-infected Huh7 cells. Infected cells were fixed after 24 hours and labeled with serially diluted immune sera. In double staining the cells were labeled with both anti-HCoV S1 (green) and anti-229E N (red) sera. Representative images show 1:200 sera dilutions with each serum sample. Rb, rabbit antiserum (panel a); Gp, guinea pig antiserum (panel b).

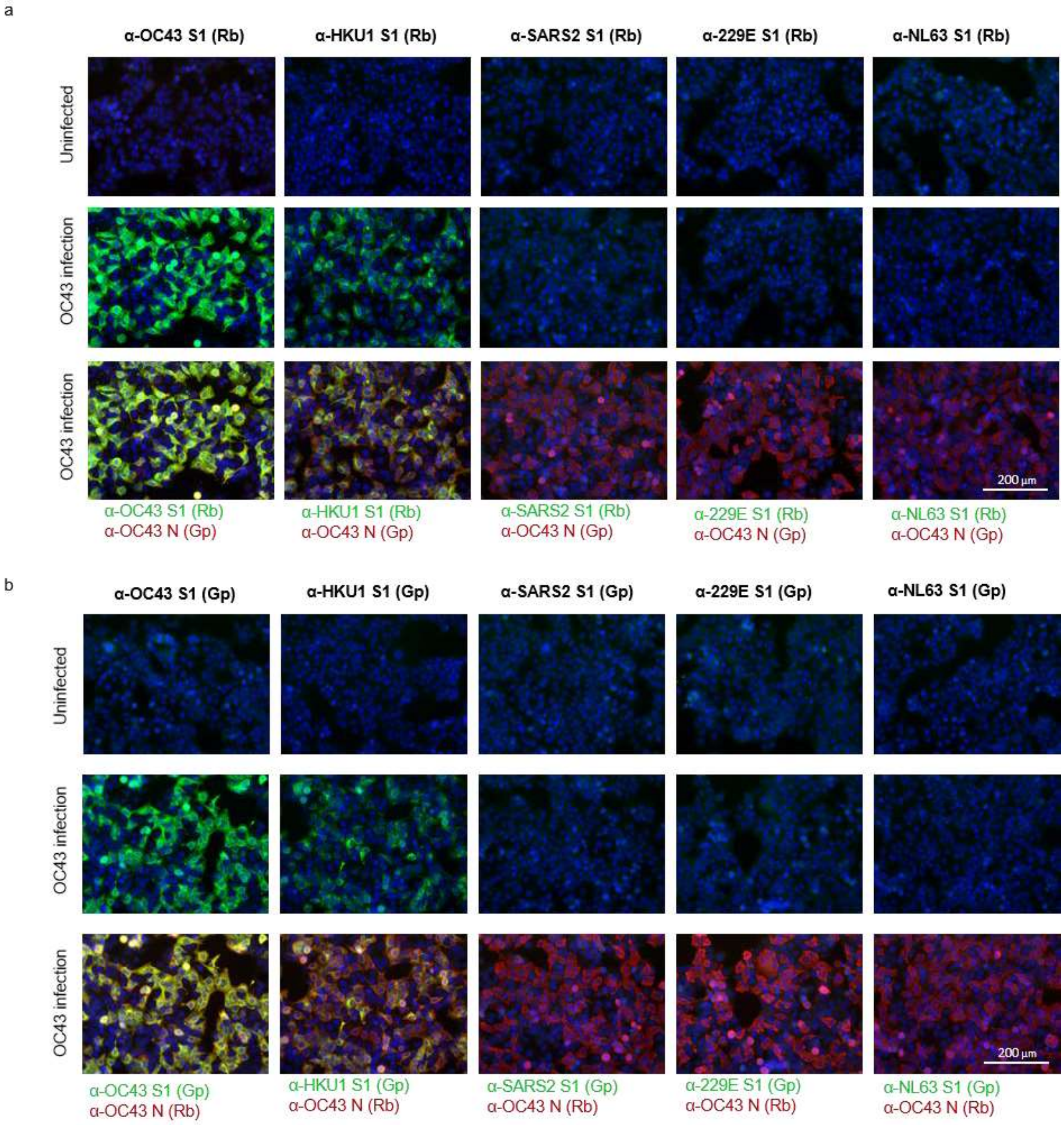

Supplementary Figure 9. Reactivity of anti-HCoV S1 sera with OC43 virus-infected cells in immunofluorescence. Reactivity of immune sera from HCoV S1 protein immunized rabbits and guinea pigs was determined in immunofluorescence assay of OC43 virus-infected Huh7 cells. Infected cells were fixed after 24 hours and labeled with serially diluted immune sera. In double staining the cells were labeled with both anti-HCoV S1 (green) and anti-OC43 N (red) sera. Representative immunofluorescence images show 1:200 sera dilutions with each serum sample. Rb, rabbit antiserum (panel a); Gp, guinea pig antiserum (panel b).

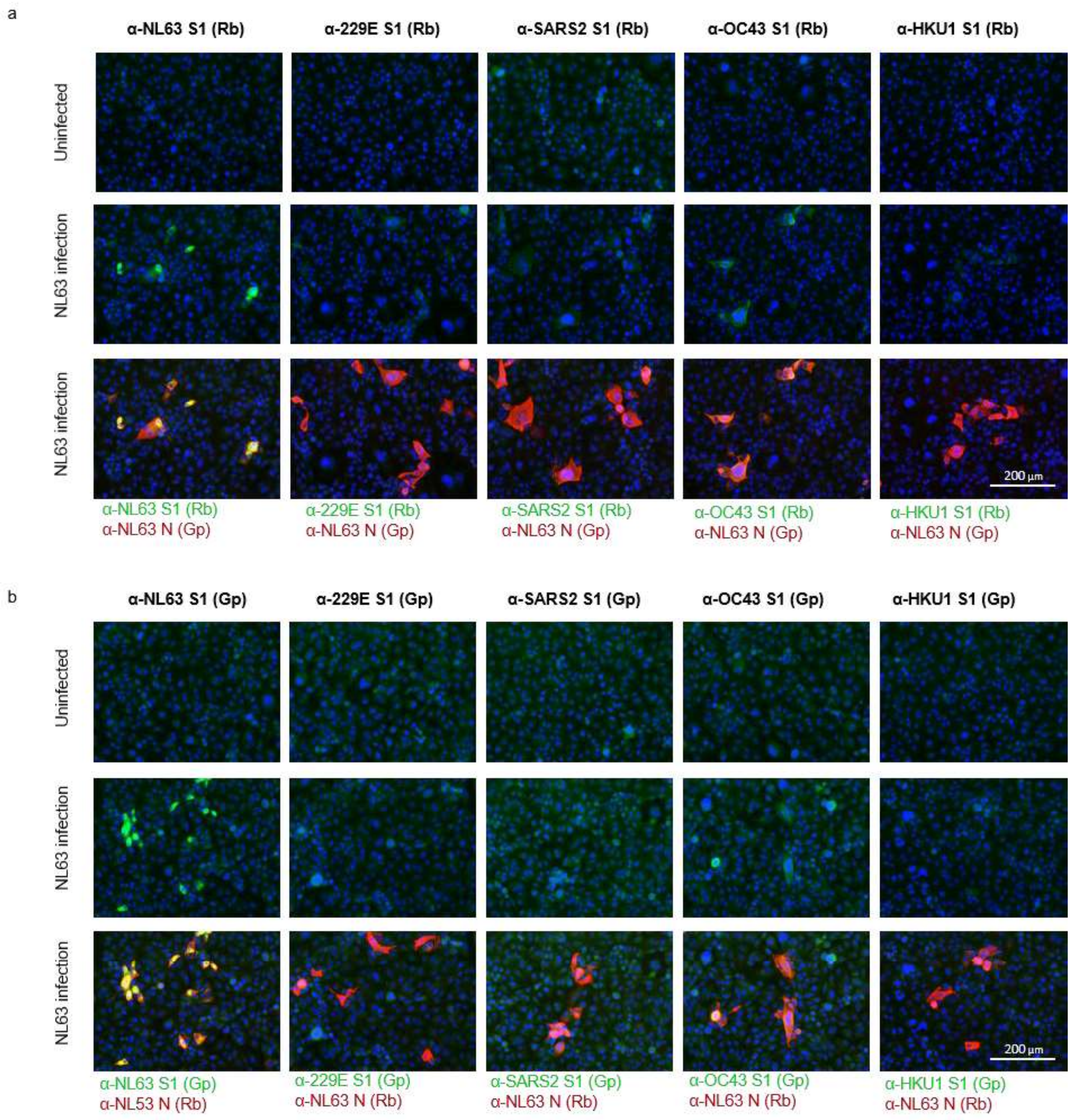

Supplementary Figure 10. Reactivity of anti-HCoV S1 sera with NL63 virus-infected cells in immunofluorescence. The titer of the reactivity of immune sera from HCoV S1 protein immunized rabbits and guinea pigs was determined in immunofluorescence assay of NL63-infected LLC-MK2 cells. Infected cells were fixed after 48 hours and labeled with serially diluted immune sera. In double staining the cells were labeled with both anti-HCoV S1 (green) and anti-NL63 N (red) sera. Representative immunofluorescence images show 1:200 sera dilutions with each serum sample. Rb, rabbit antiserum (panel a); Gp, guinea pig antiserum (panel b).

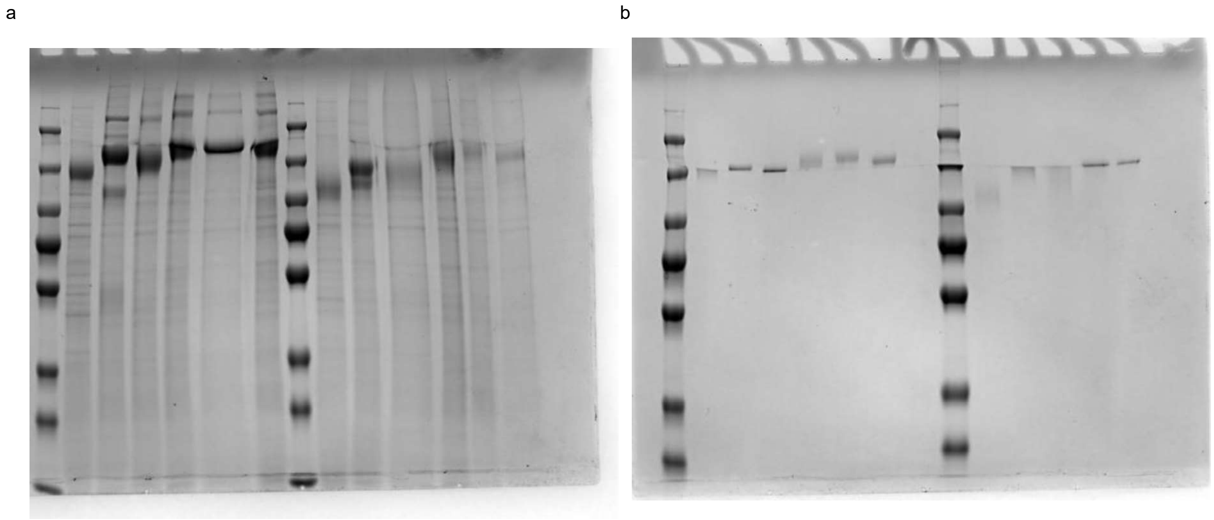

Supplementary Figure 11. Original gel images of supplementary figure 1. Coomassie stained SDS-PAGE which shows the expression of six recombinant HCoV S1 proteins in HEK293 cells. Cell suspensions (**a**) and purified proteins (**b**).
